# Cardiac complications (arrhythmias and heart failure) in patients with ischemic stroke: A meta-analysis

**DOI:** 10.1097/MD.0000000000038619

**Published:** 2024-06-21

**Authors:** Sangam Shah, Krishna Dahal, Prativa Subedi, Sangharsha Thapa, Prince Mandal, Ashutosh Kashyap, Himal Bikram Bhattarai, Ashwini Gupta, Sarita Dhakal, Sweta Singh, Swati Chand

**Affiliations:** a Tribhuvan University, Institute of Medicine, Maharajgunj, Kathmandu, Nepal; b Kist Medical College and Teaching Hospital, Imadol, Kathmandu, Nepal; c Department of Neurology, Westchester Medical Center, Valhalla, New York, USA; d Gandaki Medical College and Teaching Hospital, Pokhara, Nepal; e B P Koirala Institute of Health Sciences, Ghopa, Dharan, Nepal; f Birat Medical College and Teaching Hospital, Biratnagar, Nepal; g Department of Cardiology, Westchester Medical Center, Valhalla, New York, USA.

**Keywords:** arrythmia, complication, heart failure, ischemic, stroke

## Abstract

**Background:**

In patients with ischemic stroke (pwIS), cardiac complications have been observed in observational studies. We conducted a systematic review and meta-analysis to investigate the arrhythmias and heart failure in pwIS.

**Methods:**

Up until September 2023, we searched for case-control, cross-sectional, or cohort studies in 4 databases. For case-control/cross-sectional studies, odds ratios (OR) were determined using a random-effects model meta-analysis, while hazard ratios (HR) were calculated for cohort studies, and 95% confidence intervals (CIs) were pooled in the meta-analysis.

**Results:**

In the meta-analysis, we incorporated 5 studies: 2 cohort studies, 2 case-control studies, and 1 cross-sectional study. In all, 81,181 controls and 25,544 pwIS were included in this investigation. The combined OR for case-control studies of arrhythmias was estimated to be 1.86 (95% CI: 0.70–4.94, *P* = .21), HR for cohort studies of arrhythmias to be 4.2 (95% CI: 1.49–12.01, *P* < .05), and for cohort studies of heart failure to be 2.9 (95% CI: 2.65–3.18, *P* < .05), suggesting that pwIS may be more likely to experience cardiac complications.

**Conclusion:**

Our results revealed that there is a comparatively higher risk of cardiac complications in pwIS; however, more research is needed to evaluate the risk of cardiac complications in pwIS.

## 1. Introduction

Stroke is the second leading cause of disability as well as death worldwide, with the highest disease burden shared by low- and middle-income countries.^[1]^ In 2016, there were 13.7 million new cases of incident strokes globally, of which 87% were ischemic strokes (IS).^[1]^ In 2019, the number of stroke-related deaths was estimated to be 6.1 million.^[2]^

Cardiovascular problems following an ischemic stroke are highly prevalent, both during the acute and long-term stages.^[3]^ Within the first few days following a stroke, 10% to 20% of patients experience severe adverse cardiac events, which include a spectrum of cardiac abnormalities ranging from acute myocardial damage and coronary syndromes to heart failure or arrhythmias.^[4–10]^ The contribution of cardiac complications to the mortality of patients with stroke is variable across studies, ranging from 12.5% to 22.7%.^[5,11,12]^ Stroke patients with early severe cardiac complications are at a 2‐ to 3‐fold increased risk of short‐term mortality.^[4,13]^ However, the association of cardiac complications in patients with ischemic heart disease is not clearly demonstrated in many studies. The objective of this study was to systematically investigate the cardiac complications in ischemic stroke.

## 2. Methods

### 2.1. Study protocol

The cardiac complications (arrhythmias and heart failure) in patients with ischemic stroke (pwIS) were studied using a systematic review and meta-analysis of observational studies.^[14,15]^

### 2.2. Literature search

In September 2023, we looked for pertinent articles in MEDLINE, Embase, and the Cochrane Central Register of Controlled Trials. Cardiac complications, heart failure, arrhythmias, and ischemic stroke were used in the search method together with the Boolean operators “AND” and “OR.” We also incorporated pertinent research from review papers.

### 2.3. Study selection

The following inclusion criteria were met by the observational studies we included: the exposed/case group was made up of people with ischemic stroke, whereas the control group was made up of people without ischemic stroke; the outcome was either the odds or risk of arrhythmias or heart failure in subjects with ischemic stroke; and the study methodology was either case-control, cross-sectional, or cohort studies.

We disregarded observational studies that did not offer information on the likelihood or risk of cardiac complications (arrhythmias and heart failure) among pwIS, as well as case series lacking a control group. Two authors went through the titles and abstracts of the search results and retrieved the full texts of articles that might be eligible to see if they satisfied our criteria for inclusion. Discussions with other authors helped the 2 authors come to an agreement.

### 2.4. Risk of bias assessment

We assessed the 3 domains of selection of the studies (adequacy of the case definition, representativeness of the cases, selection of the controls, definition of the controls), comparability (comparability of cases and controls based on the design or analysis), and exposure (ascertainment of exposure, same method of ascertainment for cases and controls, non-response rate) for cross-sectional studies and case-control studies. Cohort studies were evaluated in terms of their selection, comparability (cohort comparability), and outcome (assessment of outcome, adequate length of follow-up, and adequacy of follow-up cohorts). The selection of the studies included the representativeness of the exposed cohort, selection of the non-exposed cohort, ascertainment of exposure, and demonstration that the outcome of interest was not present at the start of the study. Depending on how a study will affect the validity in each domain, it will either be graded as low risk, unknown risk, or high risk.

### 2.5. Data extraction

The initial author, publication year, nation, study design, study subjects, and study results were taken from the included studies. We divided the included studies into 2 groups: cohort studies with pwIS group and a non-IS control group, and case-control or cross-sectional studies with a pwIS group and a non-IS control group. Using the Newcastle-Ottawa Scale,^[16]^ 2 authors then independently evaluated the included studies’ risk of bias.

### 2.6. Statistical analysis

The meta-analysis was carried out with Review Manager 5.3. For case-control/cross-sectional studies, odds ratios (OR) were calculated; and hazard ratios (HR) were calculated for cohort studies. The *I*^2^ statistics were used to evaluate the heterogeneity. Significant heterogeneity is represented by an *I*^2^ value of 50%. We performed a fixed-effect model meta-analysis at an *I*^2^ value of 50%. We conducted a meta-analysis using a random-effects model if the *I*^2^ value was 50%.

## 3. Results

### 3.1. Study selection

**Figure 1.**
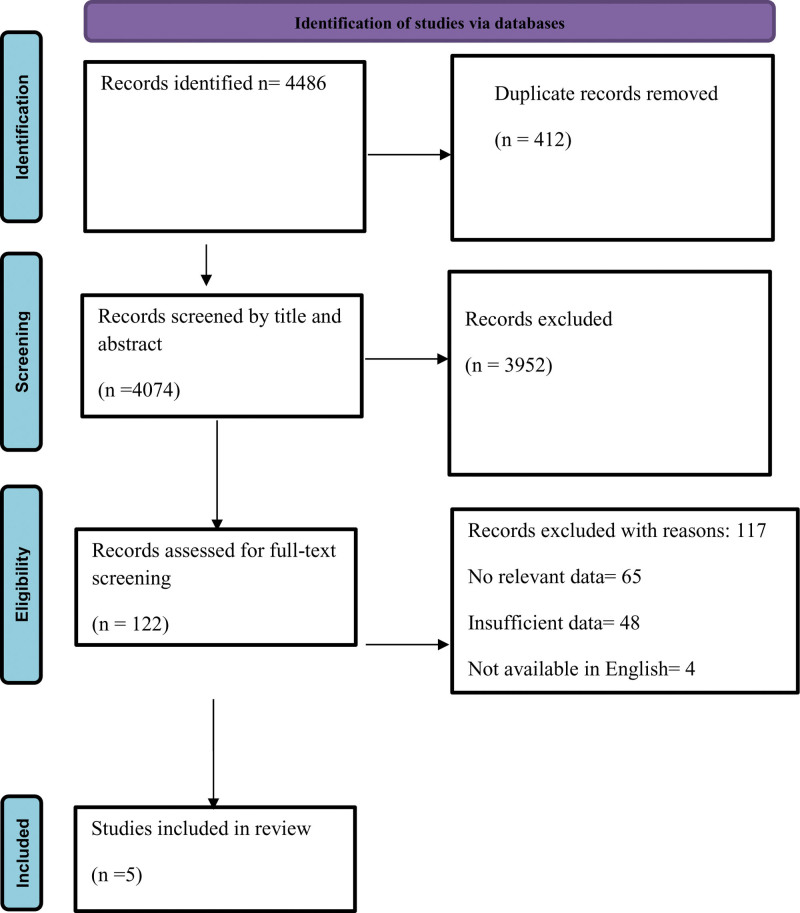
PRISMA guidelines.

A total of 4486 studies were identified and ultimately 5 studies were included in the study after duplicate removal, title and abstract screening, and finally by screening with full text. The details of the study selection are shown in Figure 1.

### 3.2. Study characteristics

There are 5 studies included in this meta-analysis whose characteristics are summarized in Table 1. Among the studies included, 2 are case-control studies, 2 are cohort studies and 1 is a cross-sectional study. The studies were conducted in Turkey, Iraq, the United States, and Canada. A total number of 81,181 controls and 25,544 pwIS were included in this study.

**Table 1:** Characteristics of included study.

Author and year of study	Study design	Sample size	Study site	Male: female
Jens Witsch et al 2018	Case-control study	2580	USA	31.8:68.2
Luciano A Sposato et al 2020	Cohort study	21,931	Canada	
Mirabela M Manea et al 2022	Case-control study	110		
Kamakshi Lakshminarayan et al 2011	Cohort study	823	USA	48.2:51.8
Osama Sukhir Muhammed Amin et al 2020	Cross-sectional study	100	Iraq	42:58

### 3.3. Risk of bias

Table 2 displays the findings of the studies’ assessment of quality. The studies were all of high quality, ranging from 8 to 9. The average result was 8.2. Sensitivity tests revealed that the aggregate estimations were not significantly affected upon removal of any 1 study at a time.

**Table 2:** Quality assessment table.

Author and year of study	Selection				Comparability	Outcomes		Total score
	Representativeness of sample	Sample size	Non-respondents/recruitment rate	Ascertainment/exposure		Assessment of outcome	Adequacy of follow-up	
Jens Witsch et al	1	1	1	2	2	1	0	8
Luciano A Sposato et al	1	1	1	2	2	1	0	8
Mirabela M Manea et al	1	1	1	2	2	1	0	8
Kamakshi Lakshminarayan et al	1	1	1	2	2	1	1	9
Osama Sukhir Muhammed Amin et al	1	1	1	2	2	1	0	8

### 3.4. Odds of arrhythmias in case-control and cross-sectional studies

**Figure 2.**
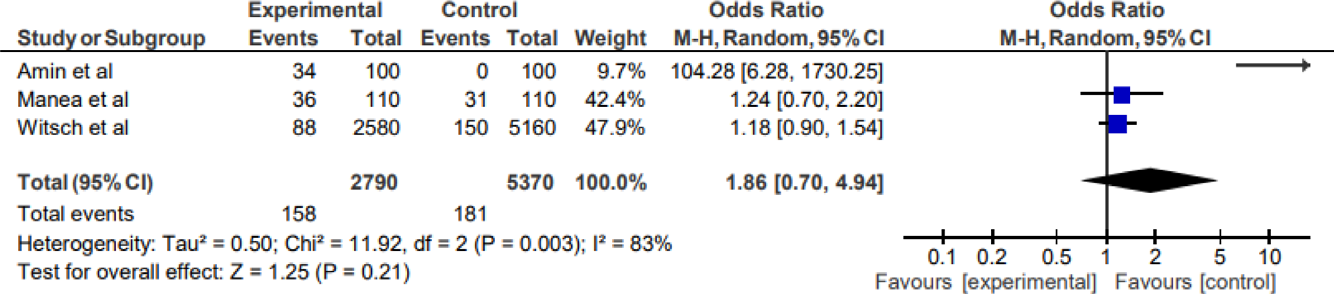
Arrhythmias in case-control and cross-sectional studies.

Among the 5 studies included, 2 were case-control studies and 1 was a cross-sectional study. In these studies, there were 2790 stroke patients and 5370 controls. As shown in Figure 2; high heterogeneity (*I*^2^ = 83%, *P* < .003) was observed, so the random-effects model was used. There is no significant increase in the prevalence of arrhythmia in stroke: pooled OR 1.86 (95% confidence interval (0.70, 4.94)), p (overall effect) = 0.21. The supplemental Figure 1, http://links.lww.com/MD/M959 shows a funnel plot showing publication bias across different studies.

### 3.5. Risk of arrhythmias in patients with stroke in cohort studies

**Figure 3.**
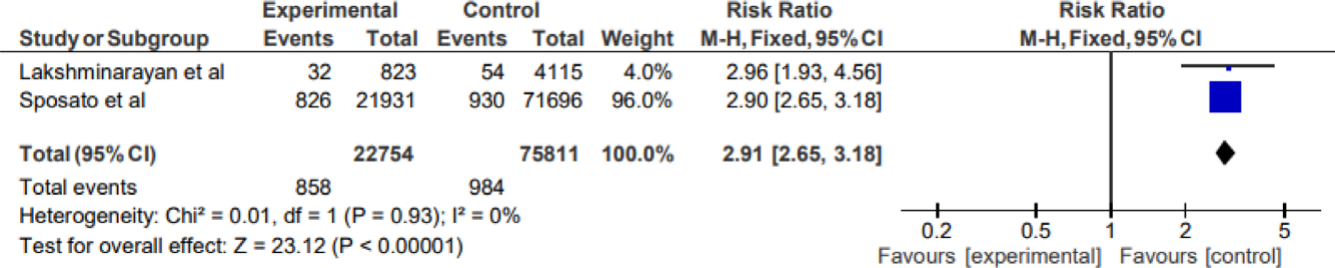
Arrhythmias in cohort studies.

We have 2 cohort studies with a total of 22,754 stroke patients and 75,811 controls. As shown in Figure 3, high heterogeneity (*I*^2^ = 98%, *P* < .00001) was observed, so the random effect model was used. The pooled HR of the 2 studies was 4.23 (95% CI: 1.49–12.01), p (overall effect) = 0.00001, suggesting that there was a significantly increased risk of arrhythmias in pwIS. The supplemental Figure 2, http://links.lww.com/MD/M960 shows a funnel plot showing publication bias across different studies.

### 3.6. Risk of heart failure in patients with stroke in cohort studies

**Figure 4.**
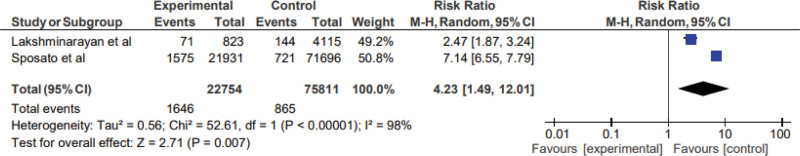
Heart failure in cohort studies.

Based on the results of 2 cohort studies, the pooled HR was 2.91 (95% CI: 2.65–3.18), p (overall effect) = 0.00001, suggesting that patients with stroke were associated with significantly increased risk of heart failure, as shown in Figure 4. Very low heterogeneity was observed (*I*^2^ = 0%, *P* < .00001), so a fixed-effect model was used in the analysis. The supplemental Figure 3, http://links.lww.com/MD/M961 shows a funnel plot showing publication bias across different studies.

## 4. Discussion

In this systematic review and meta-analysis, we studied the cardiac complications in the form of arrhythmias and heart failure in pwIS. In the meta-analysis, a pooled HR in cohort studies was significant for both arrhythmias (4.23 (95% CI: 1.49–12.01), p (overall effect) = 0.00001) and heart failure (2.91 (95% CI: 2.65–3.18), p (overall effect) = 0.00001). However, the odds ratio for arrhythmias in case-control and cross-sectional studies (pooled OR 1.86; 95% confidence interval (0.70, 4.94), p (overall effect) = 0.21) was not significant. In 2005, a systematic review had shown the risk of myocardial infarction following transient ischemic attack and ischemic stroke. However, other cardiac issues following stroke were not taken into account in the review. Additionally, since the publication of this systematic review, the management of stroke patients has undergone a significant change. These changes may have affected the incidence and mortality of cardiac complications following acute stroke. We believe this to be the first meta-analysis showing cardiac complications (arrhythmias and heart failure) in pwIS.

In recent years, there has been an increasing understanding that the relationship between IS and heart diseases extends beyond the simple fact that both conditions are impacted by classical vascular risk factors.^[3]^ The concept of a unique stroke-heart syndrome was suggested in 2018 based on the evidence from preclinical and clinical investigations showing that acute ischemic stroke can have immediate detrimental effects on the heart.^[4,6,17–19]^

Neurocardiogenic mechanisms have been suggested for heart injury caused by a stroke.^[4,6]^ Notably, neurocardiogenic injury is not only limited to cases of ischemic stroke; rather it has also been found to be associated with other acute brain illnesses like intracranial bleeding, traumatic brain injury, and seizures.^[20]^ Recent developments have contributed to a better understanding of the intricate interactions between the features of an ischemic stroke, local and systemic mediators, and the cardiac processes that follow, ultimately resulting in stroke-heart syndrome.^[3]^

There is proof that certain ischemic lesion locations increase the risk of stroke-heart syndrome. One extensively researched example is the correlation between acute myocardial damage, thrombosis with thrombocytopenia syndrome and arrhythmia and ischemic lesions in the insular cortex.^[13,21–23]^ As a fundamental component of the central autonomic network (CAN), the insular cortex has a role in efferent cardiovascular responses to emotional experiences as well as cardiac interoception.^[24,25]^ The CAN controls the outflow of sympathetic and parasympathetic neurons to the heart under physiological settings.^[25]^ An ischemic stroke may cause sudden alterations in the physiological organization of CAN, with subsequent autonomic dysfunction. Critical mediators of the stroke-heart syndrome are not well understood. Among the most frequently mentioned mechanisms of cardiac dysfunction in the stroke-heart syndrome are sympathetic hyperactivity and decreased parasympathetic activity.^[4,6,26]^

This systematic review and meta-analysis have its own limitations. There was significant heterogeneity between the studies included in this meta-analysis. We did not consider different subtypes of ischemic stroke, arrhythmias and heart failure. Similarly, risk in different ages, gender and other demographic parameters could not be clarified due to insufficient data. Further, the number of studies showing cardiac complications (arrhythmias and heart failure) in pwIS is quite limited.

## 5. Conclusion

This systematic review and meta-analysis present an association of cardiac complications in pwIS, which is of paramount importance for clinicians to consider for timely diagnosing and optimally managing these complications. However, further large-scale studies are needed to fully understand the mechanism linking cardiac complications with ischemic stroke.

## Author contributions

**Conceptualization:** Sangam Shah.

**Data curation:** Krishna Dahal, Prince Mandal, Himal Bikram Bhattarai.

**Investigation:** Sangam Shah.

**Methodology:** Sangam Shah, Krishna Dahal, Ashutosh Kashyap.

**Supervision:** Swati Chand.

**Writing – original draft:** Sangam Shah, Krishna Dahal, Prative Subedi, Sangharsha Thapa, Ashwini Gupta, Sarita Dhakal.

**Writing – review & editing:** Sangam Shah, Krishna Dahal, Prative Subedi, Sangharsha Thapa, Prince Mandal, Ashutosh Kashyap, Himal Bikram Bhattarai, Ashwini Gupta, Sarita Dhakal, Sweta Singh, Swati Chand.

## Supplementary Material






