# Supplementary figures and images for: Cardiac complications (arrhythmias and heart failure) in patients with ischemic stroke: A meta-analysis
Source: Medicine (Baltimore). 2024 Jun 21;103(25):e38619. doi: 10.1097/MD.0000000000038619 (PMC11191883; doi:10.1097/MD.0000000000038619)

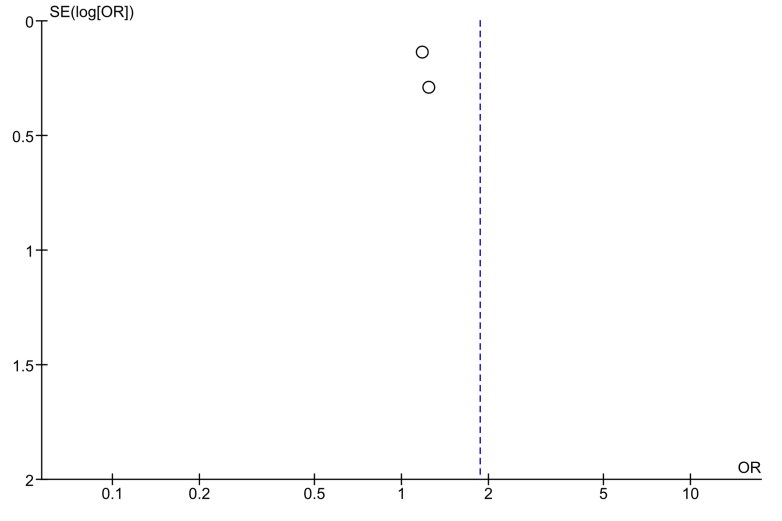


Supplemental figure 1: Funnel plot of case control and cross-sectional studies for arrythmias

Supplement: Supplementary file 1 [file medi-103-e38619-s001.docx]

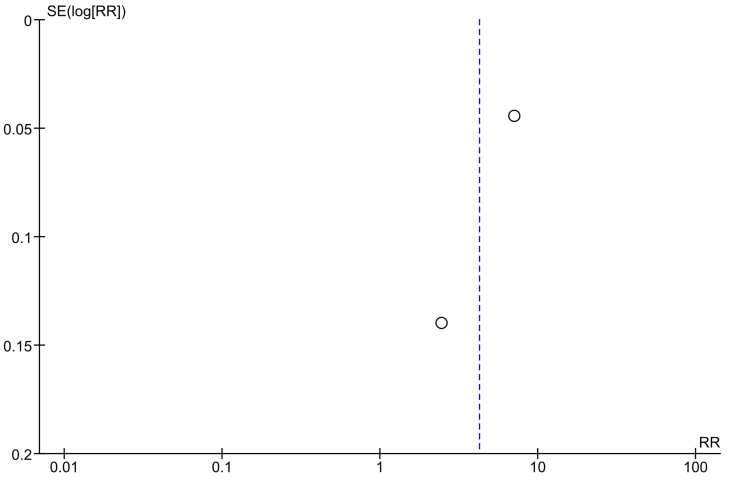


Supplemental figure 2: Funnel plot of cohort studies for arrythmias

Supplement: Supplementary file 2 [file medi-103-e38619-s002.docx]

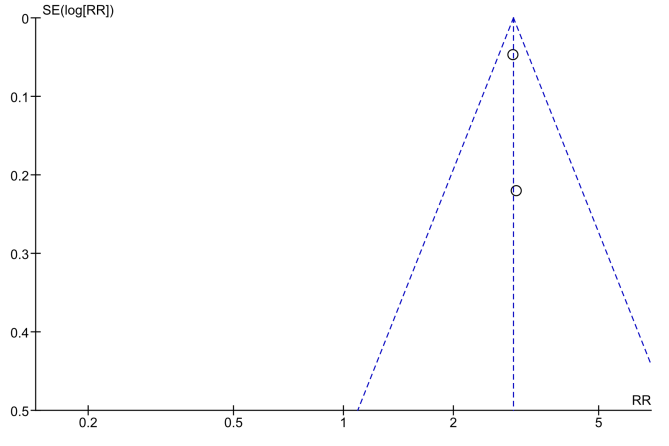


Supplemental figure 3: Funnel plot of cohort studies for heart failure

Supplement: Supplementary file 3 [file medi-103-e38619-s003.docx]
